# Light Driven Active Transition of Switching Modes in Homogeneous Oxides/Graphene Heterostructure

**DOI:** 10.1002/advs.201900213

**Published:** 2019-04-12

**Authors:** Xiaoli Chen, Kelin Zeng, Xin Zhu, Guanglong Ding, Ting Zou, Chen Zhang, Kui Zhou, Ye Zhou, Su‐Ting Han

**Affiliations:** ^1^ Shenzhen Key Laboratory of Flexible Memory Materials and Devices College of Electronic Science and Technology Shenzhen University Shenzhen Guangdong 518060 P. R. China; ^2^ Institute for Advanced Study Shenzhen University Shenzhen Guangdong 518060 P. R. China; ^3^ College of Chemistry and Environmental Engineering Shenzhen University Shenzhen Guangdong 518071 P. R. China

**Keywords:** graphene, heterostructures, homojunctions, negative differential resistance, titanium oxide

## Abstract

Depending on the mobile species involved in the resistive switching process, redox random access memories and conductive bridge random access memories are widely studied with distinct switching mechanisms. Although the two resistance switching types have faithfully proved to be electrochemically linked in metal oxide‐based memristive devices, the corresponding photo‐induced transition has not yet been realized. Here, a photo‐induced transition through the integration of a graphene layer into a titanium oxide‐based memory device is demonstrated. Coupled with Raman mapping and an electron energy loss spectroscopy technique, the photo‐induced interaction at the heterostructure of graphene/titanium oxide are considered to dominate the transition process. Moreover, a negative differential resistance effect is observed by controlling the applied voltage, which can be credited to the saturation of trap centers (oxygen vacancies) and the increase of interfacial barrier at the graphene/titanium oxide heterojunction.

Memristive devices, commonly composed of metal–insulator–metal structure, are resistance switching memories whose electrical states can be reversibly modulated by programming operation.^1^, ^2^, ^3^ Driven by the high petition for low power consumption, low cost, and high density in future information technology, memristive devices are eliciting intense investigation due to their great potential toward emerging applications for data storage, logic circuit, and neuromorphic computing.^4^, ^5^, ^6^, ^7^, ^8^, ^9^, ^10^ Although significant progress has been achieved in understanding the resistive switching process of memristive devices, challenges still exist in identifying various types of resistive switching mechanisms sometimes coexist in the same device. The oxygen vacancy filament and metal filament are highly depending on the mobile species involved in the switching process.^11^, ^12^, ^13^, ^14^

Titanium oxide (TiO*_x_*) has received particular prominence since the milestone work carried out by Hewlett‐Packard laboratory in 2008, and oxygen vacancies are widely believed to account for the resistive switching behavior both experimentally and theoretically.^15^, ^16^, ^17^ Nevertheless, the presence of a bridge between the oxygen vacancy filament and metal filament in TiO*_x_*, HfO*_x_*, and TaO*_x_* memristive devices have been confirmed by Valov and co‐workers, as the migration of cation species cannot be excluded from the switching process.^18^ Moreover, the transition from oxygen vacancy filament to metal filament have been recently demonstrated by controlling the concentration of oxygen vacancies: (i) postannealing treatment to reduce the amount of oxygen vacancies in crystal TiO_2_ memory device;^19^ (ii) introducing an interface layer (graphene or amorphous carbon) to suppress oxygen redox reactions in Ta/Ta_2_O_5_‐based memristive devices.^20^ Liu and co‐workers have recently demonstrated that the electrical characteristics of metal oxides are highly impacted by the oxygen vacancy concentration. Particularly, they proved that the switching among different electrical states can be organized by modulating the concentration of defect and a threshold value for the defect concentration was found to dictate the resistive switching behavior.^21^, ^22^, ^23^ Despite the oxygen vacancy filament and metal filament are proved to be electrochemically linked, the corresponding photo‐induced transition in metal oxide‐based memristive devices has not been reported so far.

As one of the most prevalent 2D materials, graphene has been extensively utilized in stacks of layer materials to enable various combinations of mechanical, electrical, and optical properties for the construction of multifunctional devices.^24^, ^25^, ^26^, ^27^ Photo‐induced doping of graphene could generate unique phenomena and device functionality.^28^, ^29^, ^30^, ^31^, ^32^ It has been observed that the photogenerated charges can be induced at the graphene/TiO*_x_* heterostructure, leading to a high doping level of graphene owing to the intrinsic oxygen vacancies in TiO*_x_* layer.^28^ Furthermore, when stacking with other materials, the low density of states (DOS) of graphene would reduce the efficiency of carrier transportation, resulting in a low current level which is favorable for low power information storage.^29^, ^33^

In this work, we explored the resistive switching characteristics of graphene/titanium oxide heterostructure. The homojunction of titanium oxide layers was designed to produce the rectifying resistive characteristics. The presence of graphene was proved to be able to reduce the current level through increased built‐in resistance, and to monitor the movement of oxygen vacancies under electrical field. Moreover, negative differential resistance (NDR) behaviors were witnessed in the graphene/titanium oxide devices, which is similar with previous reports including uniaxial strained graphene,^34^ graphene nanoribbon break junctions,^35^, ^36^, ^37^ and boron nitride graphene heterostructures.^38^ The saturation of traps combined with the increase of interfacial barrier during electron tunneling process were considered to dominate the NDR behavior, which hold promising perspective in implementing of critical circuit elements in electronic devices.^36^, ^37^, ^39^ Most importantly, a photo‐induced transition from oxygen vacancy filament to metal filament was observed for the first time. Through Raman mapping and electron energy loss spectroscopy (EELS) techniques, a dramatically reduction of oxygen vacancies inside the oxide layers was evidenced, which accelerate the dynamic migration of titanium cations, thus shifting the resistive switching mode to the metal filament formation.

The graphic structure of the memristive device comprising titanium oxide homojunction (TiO*_x_* and TiO*_y_*) and inserted graphene layer is shown in **Figure** **1**a, where ITO and Al were used as bottom and top electrode, respectively. Thorough device construction processes are dictated in the Experimental Section of the Supporting Information. Figure 1b presents the corresponding cross‐sectional scanning transmission electron microscopy (STEM) image of the device with silicon as the substrate. Except the compositional TiO*_x_* and TiO*_y_* layer, the formation of Al_2_O_3_ layer can also be identified due to the redox reaction between Al electrode and TiO*_y_* layer, which have been reported in other work.^17^ The thickness of TiO*_x_*, TiO*_y_*, and Al_2_O_3_ layers were respectively 20, 30, and 6 nm. Although the graphene layer cannot be detected in the cross‐sectional STEM image, the AFM characterization certifies its existence (Figure S1, Supporting Information). The graphene layer was prepared through wetting transfer technique and placed between the bottom electrode (ITO) and TiO*_y_*. For the purpose of comparison, devices without graphene layer were also fabricated as control samples. The Raman spectrum shown in Figure 1c indicates the good quality of the as transferred graphene and the integrity can be well preserved after integration into the device. The titanium oxide homojunction layers were prepared via sol–gel method since the solution processed amorphous TiO*_x_* has relatively large bandgap and already contains inherent oxygen vacancies, which is beneficial to forming free performances.^40^, ^41^ X‐ray photoelectron spectroscopy (XPS) was implemented to examine the chemical states of the constituent oxide layer before and after annealing treatment. The typical Ti 2p core level spectra located respectively at ≈465.0 eV (Ti 2p_1/2_) and ≈459.0 eV (Ti 2p_3/2_) were observed in all measured samples, as illustrated in Figure 1d,e, suggesting that the dominant species in TiO*_x_*/TiO*_y_* was Ti^4+^.^17^, ^19^ The different Ti 2p peak position of TiO*_x_*/TiO*_y_* from that of TiO*_x_* and TiO*_y_* after annealing treatment indicate that an interaction between the interface of TiO*_x_* and TiO*_y_* presumably occurred. Moreover, the color of the homojunction film turned from brown to blue after annealing treatment (Figure S2, Supporting Information), which further attested the interaction between TiO*_x_* and TiO*_y_*. As displayed in Figure 1f, the doublet O 1s peaks of TiO*_x_*, TiO*_y_*, and TiO*_x_*/TiO*_y_* centering at ≈532.2 eV (oxygen deficient) and ≈530.8 eV (Ti^4+^—O band) jointly evidence the presence of defects in the oxide layers. And the similarity of the O 1s spectra before and after postannealing treatment suggests that the concentration of oxygen vacancy were preserved after annealing treatment. This is different from the previous reports where postannealing treatment would reduce the contents of oxygen vacancy, and we assume that maintained oxygen vacancy was benefit from the existence of double layer.^19^ AFM characterization was performed to measure the surface quality of TiO*_x_*, TiO*_y_*, and TiO*_x_*/TiO*_y_* before and after annealing treatment. As illustrated in Figure 1g–i, each layer has a quite smooth surface with roughness values ranging from 0.36 to 1.11 nm, which ensures the reproducibility of memristive performance. Despite particles were observed in annealed samples due to the crystallization process, the amorphous state of TiO*_x_*/TiO*_y_* were still maintained, as demonstrated by X‐ray diffraction patterns before and after annealing treatment (Figure S3, Supporting Information) and the high‐resolution cross‐sectional STEM images (Figure S4, Supporting Information).

**Figure 1:**
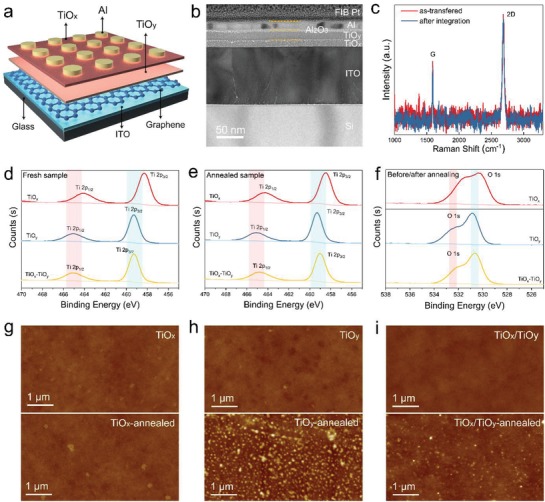
a) Schematic of the memristive device with titanium oxide homojunction (TiO*_x_* and TiO*_y_*) as functional layer, graphene as inserted electrode, ITO and Al as bottom and top electrode. b) Cross‐sectional STEM image of the device structure. c) The Raman spectra of the as transferred graphene and the graphene after depositing onto the ITO. XPS of the constituent oxide layer with d) Ti 2p core level before annealing treatment, e) Ti 2p core level after annealing treatment, f) O 1s before and after annealing treatment. AFM images of g) TiO*_x_*, h) TiO*_y_*, and i) TiO*_x_*/TiO*_y_* before and after annealing treatment.

TiO*_x_* based memristive devices have been intensively studied as oxygen vacancy filament dominated resistive switching model, where two obvious SET and RESET process can be usually observed along with the formation/rupture of conducting filaments caused by the drifting of oxygen vacancies.^42^, ^43^, ^44^, ^45^ Most relevant research works regarding to TiO*_x_* system have been summarized in Table S1 of the Supporting Information, where the devices preserve clear SET and REST processes were clarified as the resistive random access memory (RRAM) and the devices hold gradually stepwise resistive switching characteristics were clarified as the memristor. Unlike the mostly observed RRAM behavior, our device present the fingerprint of memristor characteristics, where enhanced and suppressed hysteresis loops were aroused by 15 cyclic voltage sweeps from positive and negative directions, respectively, as shown in **Figure** **2**a,b. Interestingly, a linear dependency of current on the applied voltage was observed in devices using single titanium oxide (TiO*_x_* or TiO*_y_*) as the active layer (Figure S5, Supporting Information), which is in stark contrast to the definition of memristor. That is, in the strict sense, the relationship between current and voltage in a memristor is necessarily nonlinear.^46^, ^47^ We assume that the linear *I*–*V* relationship of either TiO*_x_* or TiO*_y_* was attributed to the ohmic contact established by the oxide and electrode (graphene), since the amorphous oxide layer is filling with oxygen defects as confirmed above. As a consequence, the small nonlinear characteristic observed in TiO*_y_*‐based device in the positive direction (Figure S5, Supporting Information) might be stemmed from its slight higher crystallinity than that of TiO*_x_*, which is demonstrated in the AFM characterization (Figure 1h). This also explains the different resistive switching behaviors observed in our single titanium oxide system from that of crystalline titanium oxide‐based system, where sputtering,^17^, ^19^ atomic layer deposition^16^ or molecular beam epitaxial approach^25^ were used for preparing the active layer. It is reasonable to infer that the nonlinear hysteresis effect observed in the homojunction oxide layer system was attributed to the interfacial barrier between TiO*_x_* and TiO*_y_*, which is similar with the Ag/TiO_2_/NSTO/In system.^48^

**Figure 2:**
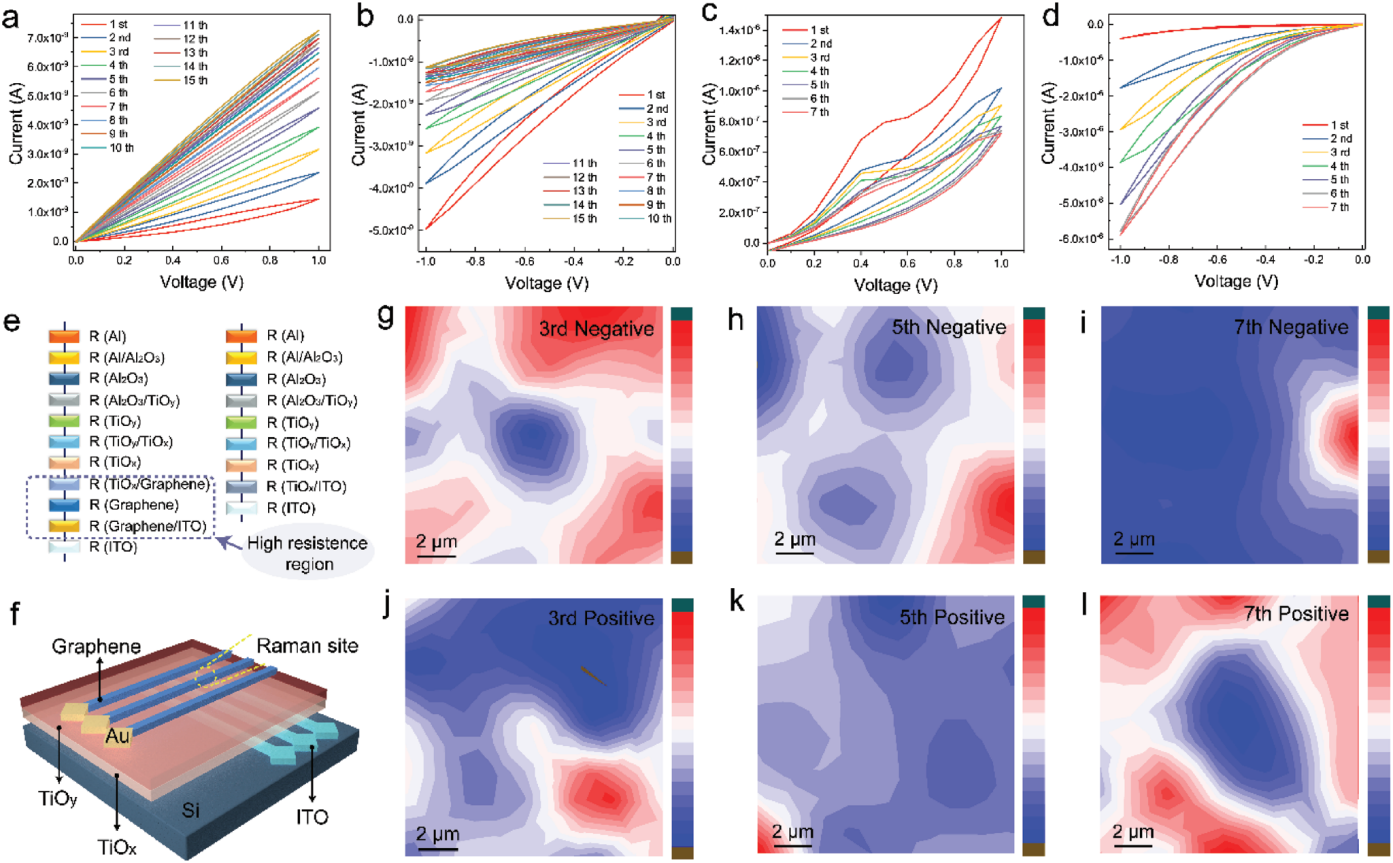
*I*–*V* characteristics of ITO/graphene/TiO*_x_*/TiO*_y_*/Al in a) positive direction, and b) negative direction. *I*–*V* characteristics of ITO/TiO*_x_*/TiO*_y_*/Al in c) positive direction, and d) negative direction. e) Schematic illustration of the series resistances between device structure with and without graphene. f) Schematic illustration of the special device with cross‐bar structure designed to pinpoint the Raman measurement sites. Raman mapping image of graphene/TiO*_x_*/TiO*_y_*/silicon with negative operation for g) three times, h) five times, i) seven times, and seven times negative operation followed by positive operation for j) three times, k) five times, and l) seven times.

Remarkably, the current level of our devices were significantly reduced compared with many other titanium oxide‐based system summarized in Table S1 of the Supporting Information, which ensures low energy consumption when integrated into the complementary metal oxide semiconductor circuit. The intrinsic high sheet resistance of the graphene should take into account for the low current level. On the other hand, on account of the low DOS of graphene near the Dirac points, it is not easy for charge carriers to tunnel through the interface, which results in high interfacial resistance between graphene and oxide heterostructure.^29^, ^49^ To verify the role of graphene, memristive devices without graphene were also fabricated and the device structure is provided in Figure S6 of the Supporting Information. Although similar memristor behavior was also observed (Figure 2d,e), the current level was three orders magnitude higher than that of the graphene inserted counterpart, which certified that the high resistance was due to the integration of graphene. The high out‐of‐plane resistance of graphene and the resistance arising from the graphene/oxide interface jointly contribute to a low current level in our device. It is commonly accepted that the general resistance of a memristor cell is comprised by the resistance of bulk of functional layers and interfaces between each layer.^50^ The schematic illustrations of the series resistances with and without graphene are shown in Figure 2e, in which the high resistance region in the device structure is circled out.

It has been well established that the resistance switching in titanium oxide‐based memristors are interrelated to the migration of oxygen vacancies. Specifically, stimulated by external forward bias, the positively charged oxygen vacancies can drift from the top to the bottom electrode, which will continuously modulate the conductance states of the memory cell. Oxygen isotope tracer technique has showed that the loss of oxygen can increase the conductivity of TiO_2_ film significantly.^16^ On opposite voltage polarity, the device will gradually relax back to its original resistance, thus leading to the hysteresis loops in positive and negative directions, resembling the consecutively potentiation and depression conductance change. However, oxygen vacancy‐based conductive filaments connecting the bottom and top electrode may not be formed duo to the high contact resistance arising from the existence of graphene. McIntyre and co‐workers found that the formation of conductive filaments was not detected when replacing electrode with ionic liquid (possess high contact resistance), whereas the electroformed conductive filaments were observed after replacing electrode with aqueous electrolyte (much more conductive).^16^ Moreover, the presentation of graphene also inhibits the formation of conductive oxygen filaments. This is supported by previous report where graphene or amorphous carbon were able to suppress oxygen redox reactions in Ta/Ta_2_O_5_‐based memristive devices.^20^ Nevertheless, in memristor systems, the dynamic movement of oxygen vacancies at various electrical states are still elusive, which make it difficult to understand the detailed resistive switching behaviors. According to Wong and co‐workers, coupled with Raman spectroscopy, graphene can be used as a robust and sensitive media to probe the changes of the underneath oxide layer.^49^ The D peak intensity of graphene in the Raman spectroscopy represents the contents of defects, which can be influenced by the interaction with oxygen vacancies. To perceive the detailed resistance switching process, Raman spectroscopy was used to mapping the profile of oxygen vacancies by characterizing the band structure of graphene. Special devices for Raman mapping were fabricated, where Al electrode was removed and the inserted graphene layer was brought to the topside acting as the top electrode, and Au patch was connected at one end of the graphene ribbon for electrical treatments. Moreover, a cross‐bar structure was applied to pinpoint the Raman measurement sites, as displayed in Figure 2f. Before conducting the Raman characterization, different Raman sites were respectively pretreated with 1 V programming operations for three, five, and seven times. As for the pretreatments corresponding to the opposite voltage sweep, first 7 times of negative bias programming operation were performed, followed by respectively three, five, and seven times of positive bias (1 V) erasing operation. Detailed diagram for the pretreatment operation of different Raman sites is provided in Figure S7 of the Supporting Information. The intensity of 2D peak was utilized to refer the concentration of oxygen vacancies, and the 2D peak intensity will be lowered with more attached oxygen vacancy. In the Raman mapping image shown in Figure 2g–l, the blue‐colored areas represent high intensity of 2D peak and the red‐colored area represent low intensity signal. The intensities of 2D peaks depict a decreased trend along with the increased sweeping times, revealing that the oxygen vacancies keep moving toward the graphene direction and result in a high concentration of defects attached to graphene. On the basis of seven times of negative programming operation, the followed positive erasing operations demonstrate an increased tendency of 2D peak intensity (Figure 2j–l), indicates that the oxygen vacancies can be drifted away from the graphene under opposite bias operation. The results can be repeatedly obtained which is well consistent with the resistance switching behavior.

**Figure** **3**a,b shows the dependency of resistance on the amplitudes of the functional bias in positive and negative directions. It is apparent that the *I*–*V* hysteresis stimulated by different amplitude of bias present remarkably different behaviors, which have potential applications in multilevel memory, thus enabling high density data storage.^51^ Notably, a prominent NDR behavior was observed when the working voltage was higher than 4 V. To be more specific, the current increased in the low voltage region (under 1.5 V), and then experienced a decreasing trend in response to the subsequently increased voltage. This voltage dependent phenomena were also applicable in the negative direction and could be repeatedly observed (Figure S8, Supporting Information), certifying that the NDR effect were not occurred randomly. The NDR effect could be utilized in many applications such as logic circuits,^52^ analog‐to‐digital converters,^53^ and high‐frequency oscillators,^54^, ^55^ and considerable explorations have been pursued into the underlying mechanism. In most studies, resonant tunneling among different localized states is considered to induce the NDR effect, either related to magnitudes of bias or the polarity. Particularly, Chi et al. have recently demonstrated that the electron trap‐assisted tunneling and the saturation of the electron traps contributed to a programmable NDR effect. The charge transport characteristics during the resistive switching process were further investigated to figure out the NDR effect occurred in our devices. We carried out the ultraviolet photoelectron spectroscopy (UPS) measurement to define the alignment of energy level of the device configuration. The evolution of HOMO onset (*E*_HOMO_) and secondary electron cutoffs (*E*_SE_) were derived from UPS spectra (Figure 3c), and the bandgaps (*E*_g_) were obtained from the Tauc plot of the absorption band position (Figure S9, Supporting Information). Correspondingly, the initial state of the energy alignment was determined as illustrated in Figure 3d, where the work function of pristine graphene and ITO were defined respectively as 4.20 and 4.85 eV. It is worth noting that the resistive switching process under electrical fields are accompanied by the charge carrier transportation, and the mass defects inside the titanium oxide layer can function as electron traps.^48^ The amount of trapped electrons can be configured by altering the magnitudes of the functional voltage. In the initial state, the charge traps (represented by straight lines) were empty, allowing the electrons to tunnel through easily and reach the graphene layer (Figure 3d). When the applied voltage is lower than 3 V, the relatively small amount of electrons can tunnel across the cell orderly without any blocking, leading to continuously increase in current. Some electrons can tunnel through the low DOS of graphene reaching to the bottom electrode, and the others contribute to a slightly n‐type doping of graphene (Figure 3e). In this process, the modulation of interface barrier between the heterostructure of graphene/oxides generated by doping are negligible, and the tunneling process will not be influenced. On the contrary, when the applied voltage is higher than 4 V, relatively large amounts of electrons are injected simultaneously to the traps, resulting in a saturation effect very soon under the high electrical field. As a result, the tunneling will be inhibited and NDR effect takes place after a small period of current increase (Figure 3f). From another perspective, the high electrical field will introduce observable n‐type doping of graphene, meanwhile the interfacial barrier at the heterojunction of graphene/oxides will be increased. Therefore, the tunneling electrons will be partially repelled, resulting in the NDR behavior.

**Figure 3:**
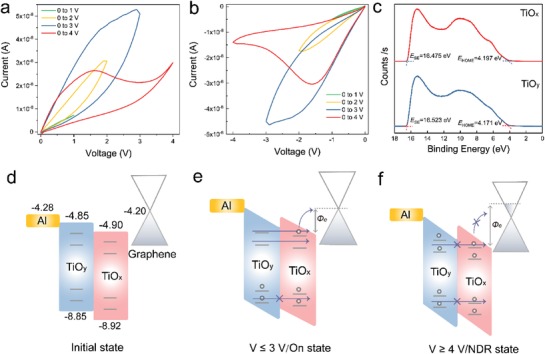
The dependence of resistance on the amplitudes of the applied bias in a) positive direction, and b) negative direction. c) UPS spectra of TiO*_x_* and TiO*_y_* with the evolution of HOMO onset (*E*_HOMO_) and secondary electron cutoffs (*E*_SE_). The energy alignments of the device structure d) in initial state, e) when the applied voltage is lower or equal to 3 V, f) when the applied voltage is higher than 4 V.

In order to further explore the functionality of our graphene/oxides heterostructure, the feature of light assisted charge transfer in graphene was investigated in this work. **Figure** **4**a shows the electrical measurement platform equipped with an installed light illumination unit (λ = 365 nm, power intensity of 3 mW cm^−2^). After 30 s of UV light illumination, the resistive switching performance undergo an apparent change where noticeable SET (*V*_SET_ = 0.58 V) and RESET (*V*_RESET_ = −0.58 V) behaviors were observed under the same electrical measurement conditions. For the purpose of comparison, the current–voltage (*I*–*V*) plots are presented in the same manner of Figure 2a,b, and the full *I*–*V* sweeping cycles are given in Figure S10 of the Supporting Information. The well repeatable current–voltage (*I*–*V*) curves illustrated in Figure 4b,c are highly consistent with the observations demonstrated by Valov and co‐workers, where they confirmed that the metallic conductive bridge were formed/ruptured during the resistive switching processes. The mobile metal cations generated from TaO*_x_*, HfO*_x_* or TiO*_x_* participated in the conductive bridge type resistance switching.^18^ Given that, we propose that conductive bridge random access memories (CBRAM)‐type memristive behaviors were dominated in our graphene/titanium oxide system after UV illumination. Different from the previous reports, the transition from oxygen vacancy filament to metal filament observed in our work is related to the light modulation, where photo‐induced light–matter interaction at the heterostructure of graphene/titanium oxides is believed to be engaged. The strong photo‐induced n‐type doping can be occurred at heterostructure of graphene/titanium oxide due to the large distributed oxygen vacancies in the TiO*_x_*.^28^ The process of photo‐induced doping is demonstrated in Figure 4d, where the defects transition, electrical transport, and charge transfer between graphene and TiO*_x_* were excited. To verify our assumption, the same Raman mapping approach as mentioned above was used. The comparison results of Raman mapping shown in Figure 4d,f demonstrate that the 2D peak intensity remarkably decreased after UV illumination, indicating that a large amount of oxygen vacancies were absorbed or even covalent bonded to the graphene layer. Therefore, the titanium cations (mostly Ti ^4+^) became the majority mobile species inside the oxide layer and the CBRAM‐type behavior started to dominate the resistive switching process.

**Figure 4:**
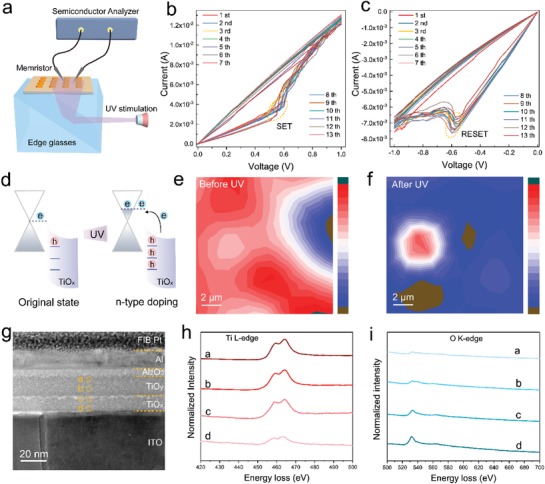
a) The electrical measurement platform equipped with a light illumination unit (λ = 365 nm, power intensity of 3 mW cm^−2^). *I*–*V* characteristics of ITO/graphene/TiO*_x_*/TiO*_y_*/Al after UV illumination in b) positive direction, and c) negative direction. d) The process of photo‐induced doping at the heterostructure of graphene/titanium oxide. The Raman mapping images of graphene e) before and f) after UV illumination. g) Cross‐sectional STEM image of the device structure labeled with four sequential measurement points. EELS spectra of h) Ti L‐edge, and i) O K‐edge in the four labeled points.

To further investigate the ionic drifting process inside the homojunction titanium oxides after light modulation, EELS in STEM was conducted. For EELS measurement, the device was pretreated with 30 s of UV light illumination, followed by programming operation with positive bias executed on top electrode. Figure 4g labels the four sequential measurement points. Although the Ti L_3_ peak (≈459 eV) can be detected across the entire region, it shows a gradual decrease trend from point a to d, attributing to the reduction of Ti^4+^ and corresponding increase of reduced Ti state (Figure 4h).^11^ This observation is highly consistent with the change of oxygen vacancy concentration reflected by the O K‐edge spectra in the corresponding measurement point. As suggested in Figure 4i, only small amount of oxygen vacancies can be detected near the top electrode, while accumulating in the vicinity of graphene/oxides interface. This phenomenon certifies the fact that oxygen vacancies tend to be bonded with graphene after UV illumination.

In light of the above findings, we propose a model to describe the competitive resistive switching behaviors before and after the light regulation. Initially, more oxygen vacancies are abounded in the oxide layer than that of the Ti^4+^. It has been widely acknowledged that oxygen vacancy is more mobile than metal cation under normal circumstances.^24^ Upon applying the forward bias on the top electrode, oxygen vacancies start to migration toward the bottom direction under electrical field, which will increase the conductivity of the cell. However, under persistent forward biasing, conductive bridge stemmed from oxygen vacancies cannot be formed owing to the high contact resistance of graphene. Instead, the moving oxygen vacancies are prone to be trapped at the interface of graphene/oxides, as have been evidenced experimentally in previous literatures.^28^ When opposite bias is executed on the top electrode, the weakly absorbed oxygen vacancies on graphene and the Ti^4+^ species will drift back, thus leading to an increase of resistance in the active layer. Whereas, stimulated by the UV illumination, interactions between oxygen vacancies and graphene are enabled and covalent bonds are considered to be formed. As a consequence, the movement of oxygen vacancy will be suppressed and Ti^4+^ become the majority species which are migrating toward the bottom direction. Then, the mobile Ti^4+^ species will be reduced at the bottom electrode and form several nuclei of Ti atom under constant forward biasing. Subsequently, the nuclei of Ti atom grow and form conductive bridge, setting the device to the low resistance state. On opposite bias, the Ti‐based conductive bridge will be damaged at the top interface and both oxygen vacancies and titanium cations migrate upward, which constitute the RESET process.

In summary, by integrating of graphene into titanium oxide homojunction‐based memristive device configuration, we demonstrated a photo‐induced transition between two competitive resistive switching modes. Before illuminating with UV light, the migration of oxygen vacancies under electrical field continuously altered the conductivity of the oxide layers, therefore oxygen vacancy filament was dominated. Once covalent bonding between oxygen vacancies and graphene was enabled by light excitation, Ti^4+^ became the majority mobile species, and metallic conductive bridge were prone to be formed. The inserted graphene was demonstrated to play two different roles: (1) reduce the current level by providing interfacial resistance (graphene/oxides); (2) monitor the movement of oxygen vacancies by Raman mapping. Moreover, multilevel conduction states were enabled by applying different amplitude of voltages, and NDR characteristic was observed by increasing the applied voltage higher than a critical value. These findings observed in our work offer fundamentally insight in designing and manipulating of memristive devices based on heterostructured materials.

## Conflict of Interest

The authors declare no conflict of interest.

## Supporting information

Supplementary
