# Supplementary material for: Light Driven Active Transition of Switching Modes in Homogeneous Oxides/Graphene Heterostructure
Source: Adv Sci (Weinh). 2019 Apr 12;6(11):1900213. doi: 10.1002/advs.201900213 (PMC6548956; doi:10.1002/advs.201900213)
Supplement: Supplementary file 1 — Supplementary [file ADVS-6-1900213-s001.pdf]

Copyright WILEY-VCH Verlag GmbH & Co. KGaA, 69469 Weinheim, Germany, 2019.

ADVANCED SCIENCE
Open Access

Supporting Information

for *Adv. Sci.,* DOI: 10.1002/advs.201900213

Light Driven Active Transition of Switching Modes in Homogeneous Oxides/Graphene Heterostructure

Xiaoli Chen, Kelin Zeng, Xin Zhu, Guanglong Ding, Ting Zou, Chen Zhang, Kui Zhou, Ye Zhou, and Su-Ting Han**

Copyright WILEY-VCH Verlag GmbH & Co. KGaA, 69469 Weinheim, Germany, 2019.

Supporting Information

Light Driven Active Transition of Switching Modes in Homogeneous Oxides/Graphene Heterostructure

Xiaoli Chen, Kelin Zeng, Xin Zhu, Guanglong Ding, Ting Zou, Chen Zhang, Kui Zhou, Ye Zhou and Su-Ting Han**

Dr. X. Chen, Dr. G. Ding, Dr. C. Zhang, Dr. K. Zhou, Prof. S.-T. Han
Shenzhen Key Laboratory of Flexible Memory Materials and Devices, College of Electronic Science and Technology, Shenzhen University, Shenzhen, Guangdong 518060, P. R. China. E-mail: sutinghan@szu.edu.cn

K. Zeng, X. Zhu, Prof. Y. Zhou
Institute for Advanced Study, Shenzhen University, Shenzhen, Guangdong 518060, P. R. China. E-mail: yezhou@szu.edu.cn

T. Zou
College of Chemistry and Environmental Engineering, Shenzhen University, Shenzhen, Guangdong 518071, P. R. China.

Keywords: Heterostructure; homojunction; titanium oxide; graphene; negative differential resistance.

Experimental Section

Synthesis of TiO_x and TiO_y : Ethanolamine ($H_2NCH_2CH_2OH$, Acros, 99%, 1 mL) and 2-methoxyethanol ($CH_3OCH_2CH_2OH$, Acros, 99+%, 10 mL) were mixed in a flask and stirred for 10 min. After that, Titanium (IV) isopropoxide ($Ti[OCH(CH_3)_2]_4$, Acros, 98+%, 1 mL) was added into the mixture. Then, for TiO_x, the temperature was raised and kept at 80°C for 24 h. Subsequently, the obtained TiO_x solution was cooled down to room temperature, meanwhile the color of solution changed to dark red. Whereas, for the synthesis of TiO_y, the difference lies in the different baking condition, and 80°C for 1 h was used for the preparation of TiO_y. After cooling down, the color of TiO_y turns into much light yellow. The original concentration of both as-synthesized precursors were 125 $mg \cdot mL^{-1}$. As 40 $mg \cdot mL^{-1}$ was the target concentration, further diluting with n-butanol ($CH_3CH_2CH_2CH_2OH$, Acros, 99%) was carried out before using.

Device Fabrication: The glass substrate (200 nm thick, 20 mm wide) patterned with transparent indium tin oxide (ITO) was cleaned with Decon 90 and deionized water sequentially in the ultrasound system. Then, graphene on copper sheet (15 mm wide) was transferred onto the glass/ITO substrate utilizing a mediator-assisted transfer method (graphene wet transfer process), connecting with ITO as the inserted electrode. After that, the diluted TiO_x solution (40 mg·mL-1) was deposited by spin-coating approach (3000 rpm for 40 s), and then annealing sequentially at room temperature (2 h) and 120 °C (2 h) were performed in nitrogen condition. Subsequently, the exactly same deposition and annealing procedures were applied for TiO_y. Finally, 30 nm of Al was deposited as top electrode through thermal evaporation method.

With respect to the control devices, including ITO/TiO_x/TiO_y/Al, ITO/graphene/TiO_x/Al and ITO/ graphene/TiO_y/Al, the process of preparing the corresponding layer was intentionally removed (such as graphene, TiO_y and TiO_y). Regarding to the special devices for Raman measurements, the graphene layer was deliberately lifted to the top layer acting as the

top electrode, and Au patch were deposited at one end of the graphene for the convenience of electrical measurement. Furthermore, for the sake of preparing of STEM and EELS samples with focus ion beam (FIB) technique, the glass substrate was replaced with silicon.

Electrical measurements and characterizations: The current-voltage (I-V) characteristics of the memristive devices were analyzed by Keysight B1500A parameter analyzer and Keysight B2902A precision source/measure unit. Voltage bias was always applied to the top Al electrodes, in the meantime, the bottom graphene electrodes was connected with ITO which was kept grounded by PRCBE probe station in ambient atmosphere. The surface morphology of the graphene and TiO_x/TiO_y film were characterized by atomic force microscope (AFM, Bruker Dimension Icon, ScanAsyst mode, ScanAsyst-Air). The cross-sectional image of the device was obtained through scanning transmission electron microscopy (STEM) (FEI Tecnai F20). Raman spectroscopy measurements were conducted using a laser with wavelength of 532 nm (LabRAM HR Evolution, Horiba scientific). X-ray powder diffraction (XRD) measurement of TiO_x and TiO_y were performed with copper K wavelength of Cu/K-α1 (1.54059 Å) (Bruker Dimension Icon, D8 discover). Ultraviolet photoelectron spectroscopy (UPS) and X-ray photoelectron spectroscopy (XPS) measurements were respectively performed on ESCALAB 250XI (Thermo Scientific) with 21.2 eV of He I and 1486.6 eV of Al Kα monochromatic source.

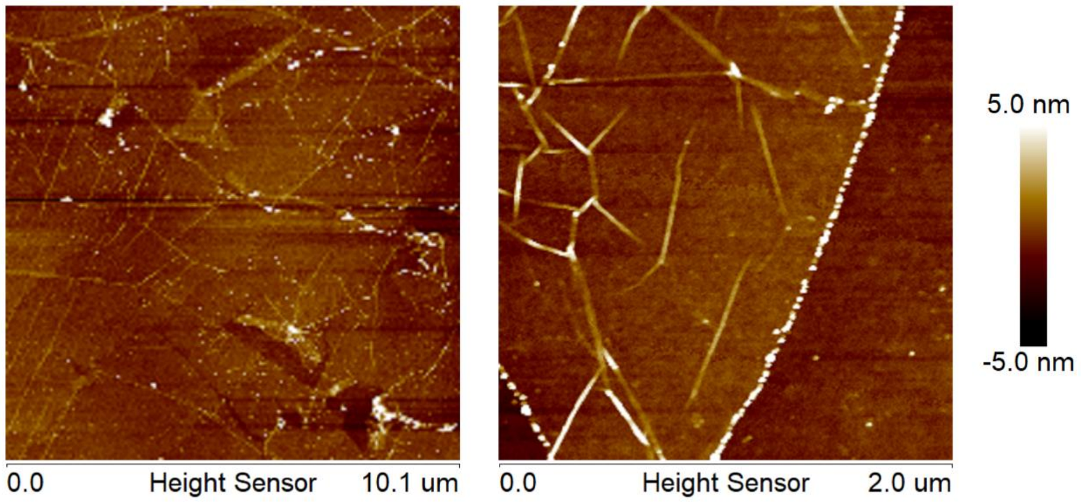

Figure S1. AFM characterization certifies the existence of graphene layer. The left represents a big area of graphene, and the right represents one edge of a piece of graphene. The bright dots (white) at the edges of graphene are the polymer residues.

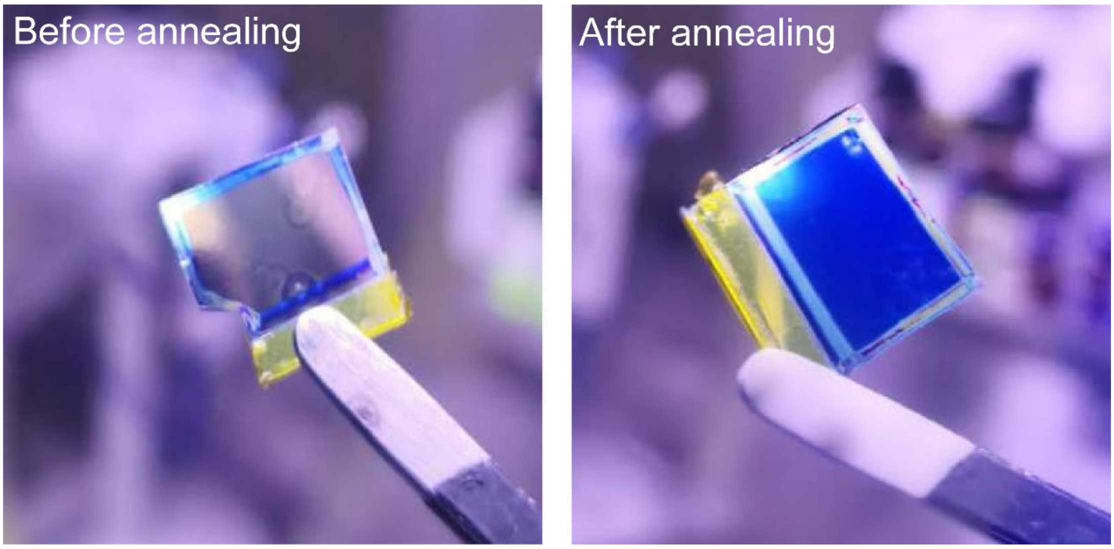

Figure S2. Images of TiO_x/TiO_y depositing on silicon substrates. The color change before and after annealing treatment indicates that interaction might be occurred between TiO_x and TiO_y at the interface, since there is no color change with either TiO_x or TiO_y before and after annealing.

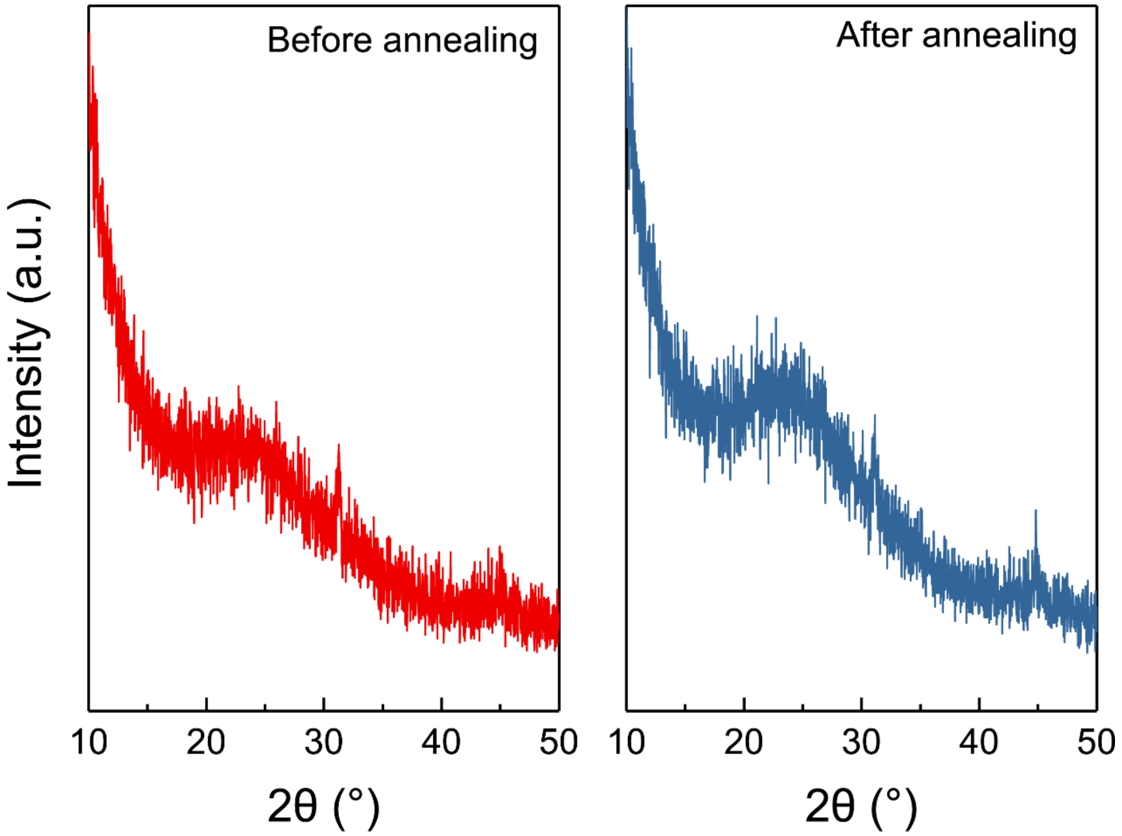

Figure S3. X-ray diffraction patterns demonstrates that the amorphous state of TiO_x/TiO_y were maintained before and after annealing treatment.

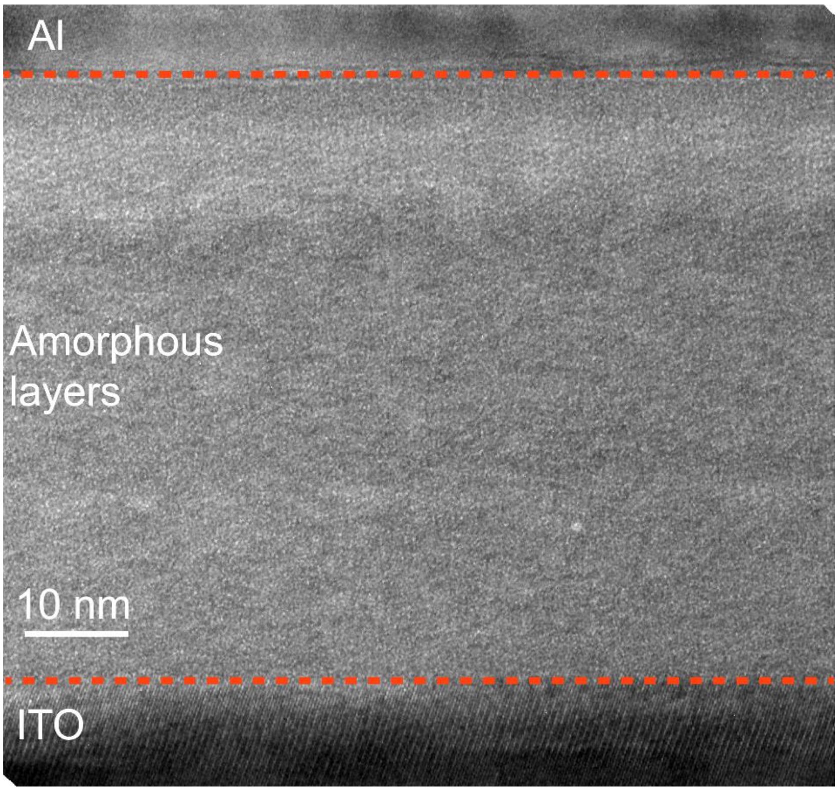

Figure S4. High-resolution cross-sectional STEM image of the device cell suggests the amorphous state of the oxide layers after annealing treatment.

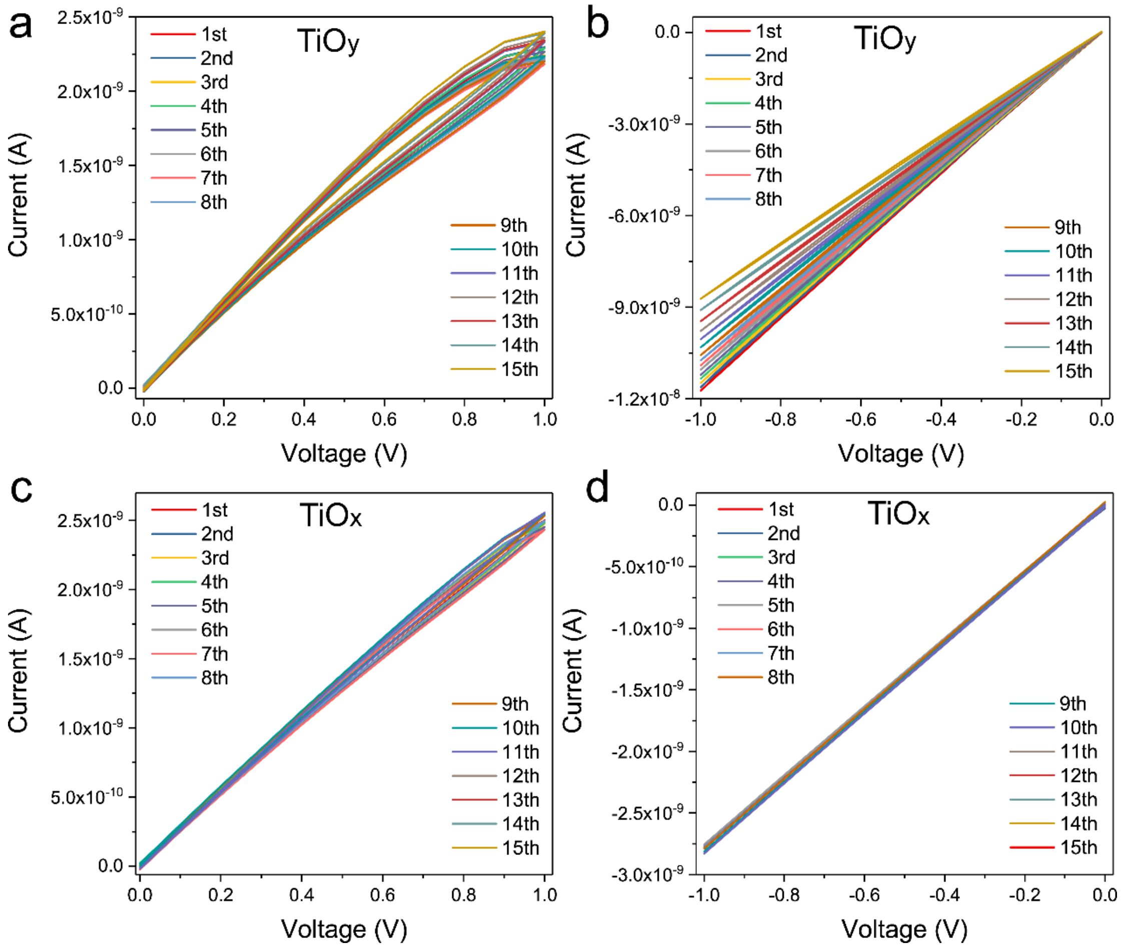

Figure S5. The linear dependency of current on the applied voltage observed in devices using single titanium oxide (TiO_x or TiO_y) as the active layer. The small non-linear characteristic observed in TiO_y-based device in the positive direction might be stemmed from its slight higher crystallinity than that of TiO_x.

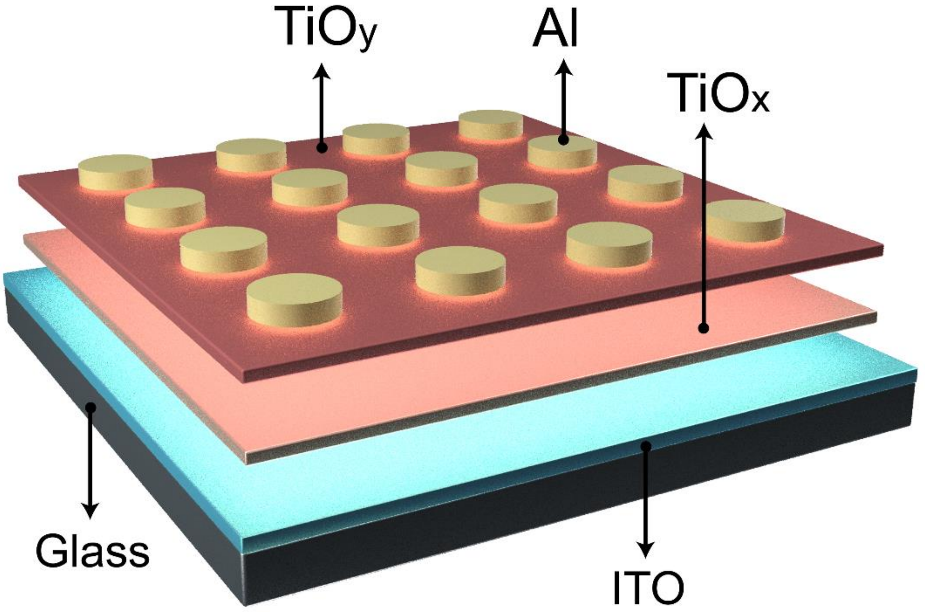

Figure S6. Memristive device structure without graphene.

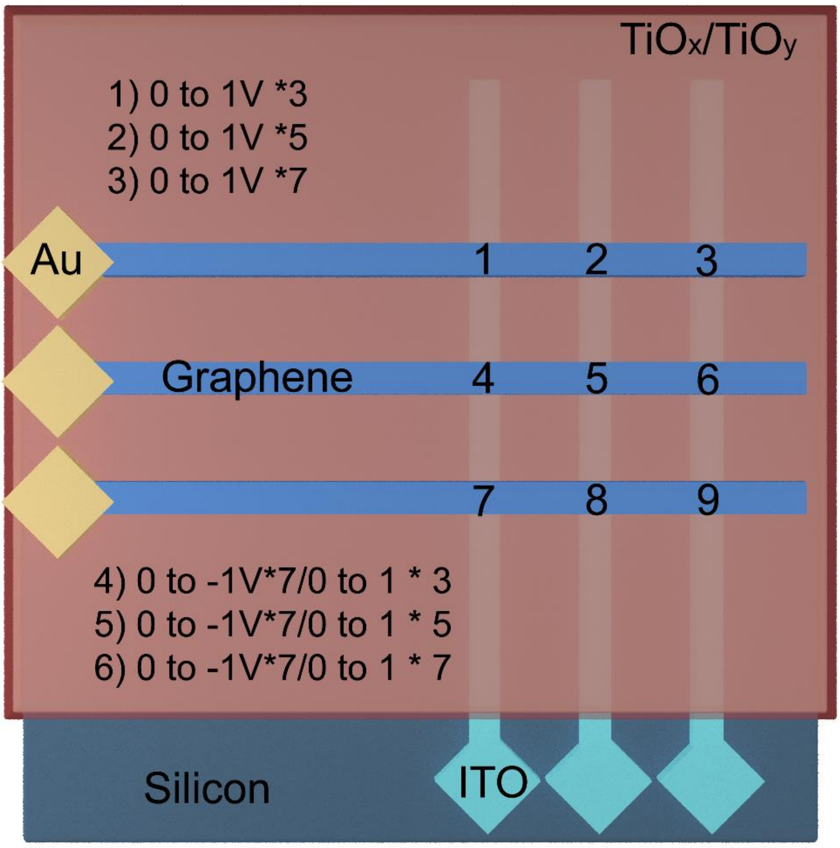

Figure S7. Detailed diagram for the pre-treatment operation of different Raman sites. For the Raman sites 7, 8, 9 and more sites on other devices were used for repeating the 1), 2), 3), 4), 5), and 6) operations to get reproducible results.

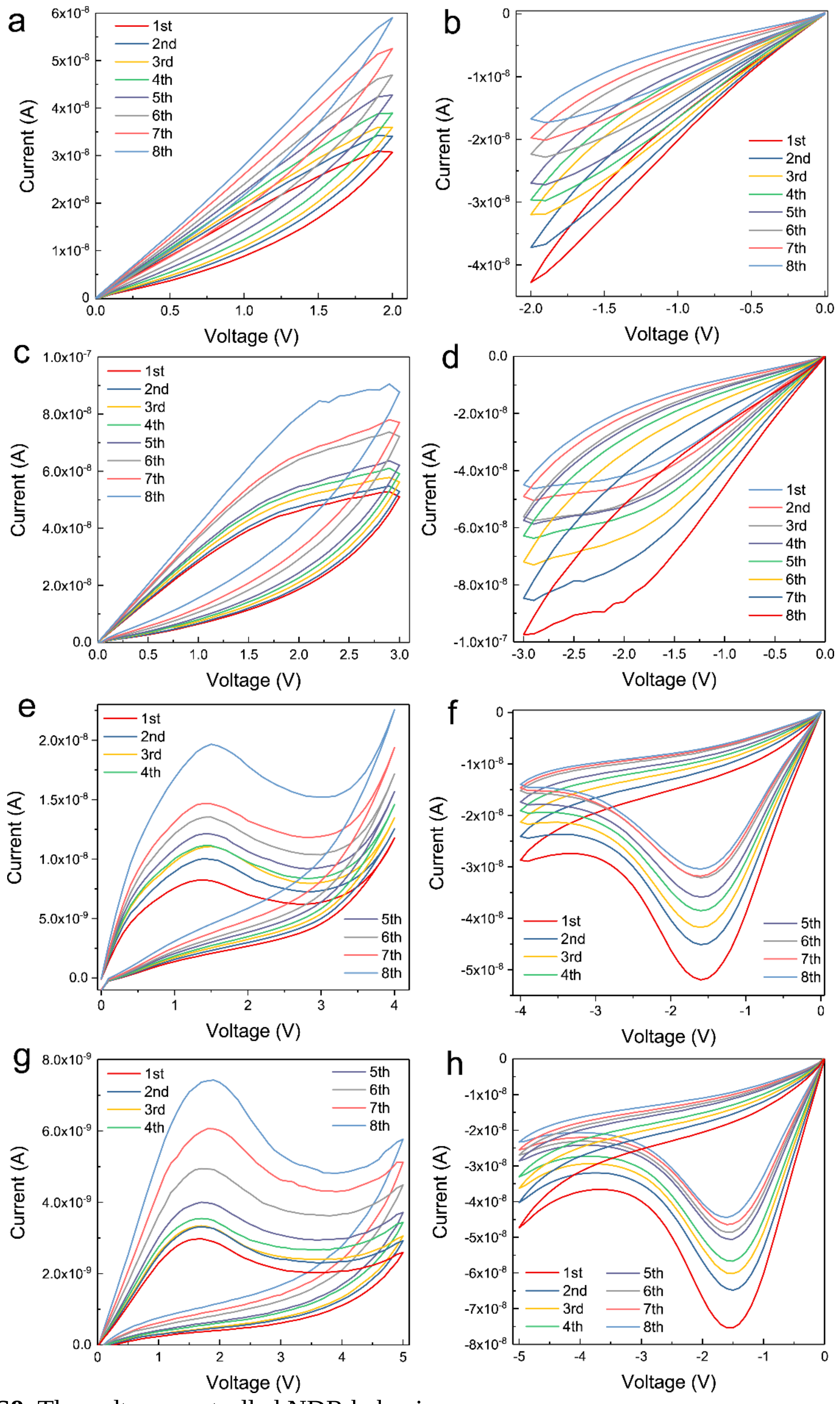

Figure S8. The voltage controlled NDR behaviors.

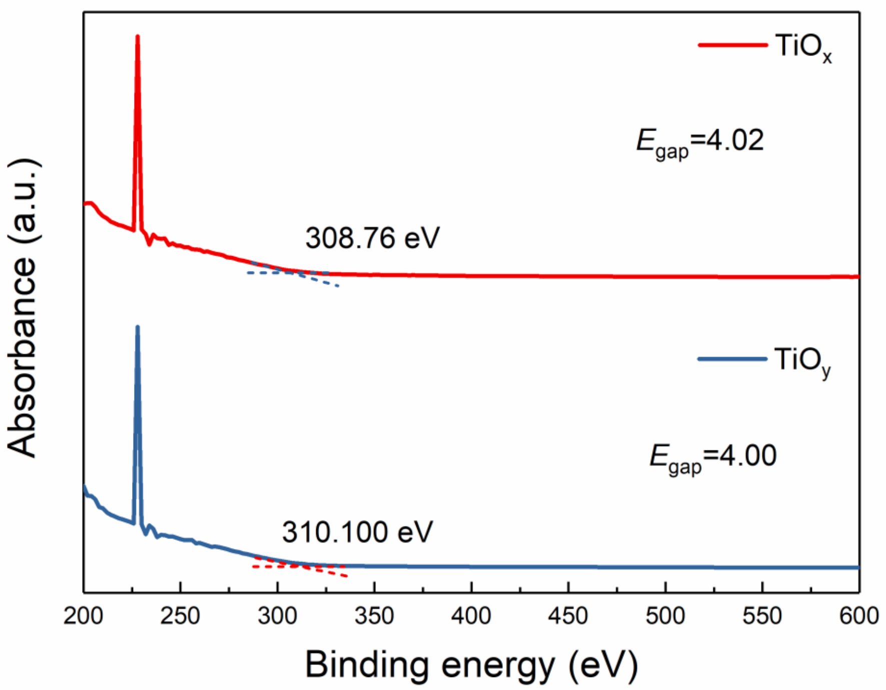

Figure S9. Band gaps (*E*g) of TiO_x and TiO_y obtained from the Tauc plot of the absorption band position.

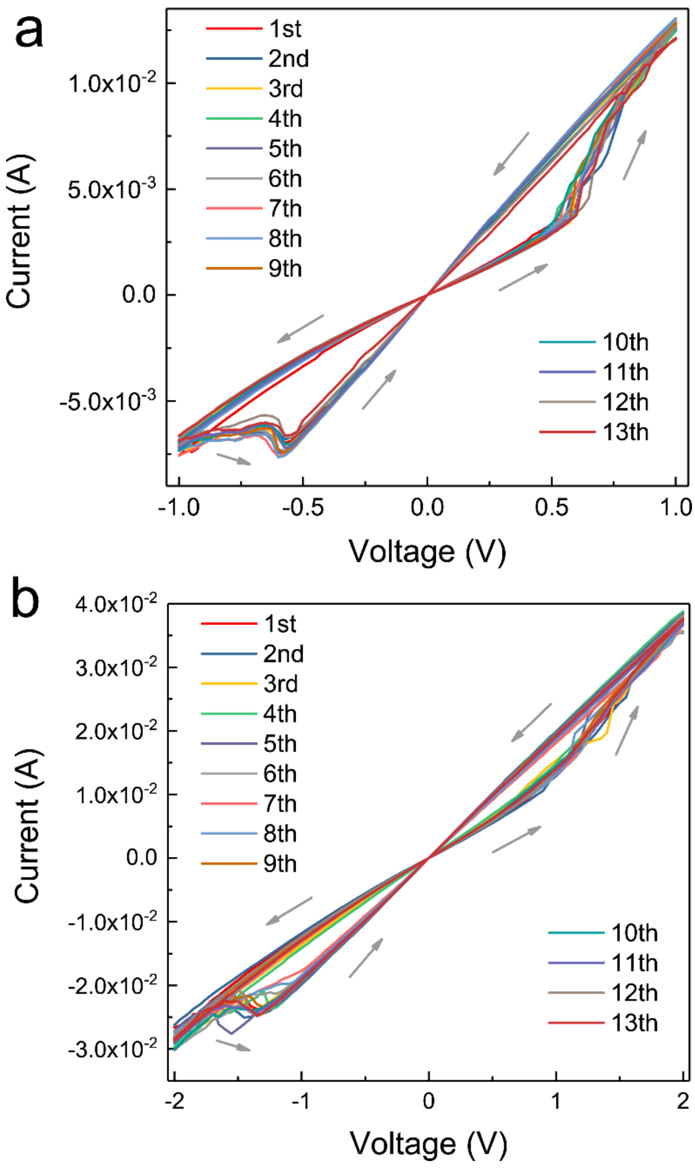

Figure S10. I-V characteristics of ITO/graphene/TiO_x/TiO_y/Al after UV illumination with voltage sweeping range from a) -1 to 1 V, and b) -2 to 2 V.

Table S1. The summary of titanium oxide-based memristive systems.

Device structure	Resistive switching characteristics	Current level (ON/OFF current)	Range of voltage
Graphene/TiO_2/Ti/Pt [1]	*RRAM*	$10^{-2}/10^{-4}$	0—4V
Ta/TaOx/TiO_2/Ti [2]	RRAM	10^{-5}	0—-5V
ITO/GO-TiO_2/Al [3]	RRAM	$10^{-3}/10^{-4}$	0—3V
Ti/Pt/TiO_2/Pt [4]	RRAM	$10^{-2}/10^{-3}$	0—2V
Pt/TiO_2/Pt [5]	RRAM	10^{-2}	0—2V
Au/Ti/TiO_2/$SrTiO_3$ [6]	RRAM	$10^{-3}/10^{2}$	0—3V
Al/TiO_x/TiO_2/AlO_x/Al [7]	*RRAM*	$10^{-4}/10^{-5}$	0—3V
Au/TiO_2/Au [8]	RRAM	$10^{-3}/10^{-5}$	0—2.5V
Al/TiO_2/Al [9]	RRAM	10^{-4}	0—3V
Al/TiO_2/FTO [10]	RRAM	10^{-3}	0—0.5V
Ag/TiO_2/Nb:$SrTiO_3$/In [11]	RRAM	10^{-2}	0—-5V
Pt/Ni/a-TiO_2/Al_2O_3/Pt [12]	RRAM	10^{-2}	0—4V
Al/TiO_2/Al [13]	memristor	10^{-8}	0—6V
Al/Sb:SnO_2/TiO_2/Al [14]	RRAM	10^{-2}	0—4.5V
Ag/TiO_2/Nb:$SrTiO_3$/Ag [15]	RRAM	10^{-2}	0—-5V
Pt/TiO_2/Pd [16]	RRAM	$10^{-2}/10^{-3}$	0—0.5V
Pt/ TiO_2/Pt/Cr [17]	RRAM	10^{-5}	0—3V
TE/TiO_2/Nb-$SrTiO_3$/BE [18]	RRAM	10^{-2}	0—2.5V
Ti/TiO_2/SiO_x/n-Si [19]	RRAM	10^{-2}	0—9V
**Al/TiO_y/TiO_x/graphene/ITO (our work)**	**memristor**	**10^{-9}**	**0—5V**

References

[1] M. Qian, Y. Pan, F. Liu, M. Wang, H. Shen, D. He, B. Wang, Y. Shi, F. Miao and X. Wang, *Adv Mater.* **2014**, *26*, 3275.

[2] Y. -F. Wang, Y. -C. Lin, I. -T. Wang, T.-P. Lin, T. -H. Hou, *Scientific Reports*, **2015**, *5*, 10150.

[3] X. Zhao, Z. Wang, Y. Xie, H. Xu, J. Zhu, X. Zhang, W. Liu, G. Yang, J. Ma and Y. Liu, *Small.* **2018**, e1801325.

[4] V. Prusakova, C. Collini, M. Nardi, R. Tatti, L. Lunelli, L. Vanzetti, L. Lorenzelli, G. Baldi, A. Chiappini, A. Chiasera, D. Ristic, R. Verucchi, M. Bortolotti and S. Dirè, *RSC Advances.* **2017**, *7*, 1654.

[5] D. H. Kwon, K. M. Kim, J. H. Jang, J. M. Jeon, M. H. Lee, G. H. Kim, X. S. Li, G. S. Park, B. Lee, S. Han, M. Kim and C. S. Hwang, *Nat Nanotechnol.* **2010**, *5*, 148.

[6] C. Hu, M. D. McDaniel, A. Posadas, A. A. Demkov, J. G. Ekerdt and E. T. Yu, *Nano Lett.* **2014**, *14*, 4360.

[7] D. Son, J. Lee, S. Qiao, R. Ghaffari, J. Kim, J. E. Lee, C. Song, S. J. Kim, D. J. Lee, S. W. Jun, S. Yang, M. Park, J. Shin, K. Do, M. Lee, K. Kang, C. S. Hwang, N. Lu, T. Hyeon and D. H. Kim, *Nat Nanotechnol.* **2014**, *9*, 397.

[8] H. A. Bafrani, M. Ebrahimi, S. B. Shouraki and A. Z. Moshfegh, *Nanotechnology.* **2018**, *29*, 015205.

[9] H. Y. Jeong, Y. I. Kim, J. Y. Lee and S. Y. Choi, *Nanotechnology.* **2010**, *21*, 115203.

[10] T. D. Dongale, N. D. Desai, K. V. Khot, N. B. Mullani, P. S. Pawar, R. S. Tikke, V. B. Patil, P. P. Waifalkar, P. B. Patil, R. K. Kamat, P. S. Patil and P. N. Bhosale, *Journal of Solid State Electrochemistry.* **2016**, *21*, 2753.

[11] Y. Zhu, M. Li, H. Zhou, Z. Hu, X. Liu, X. Fang, B. Sebo, G. Fang and X. Zhao, *Journal of Physics D: Applied Physics.* **2012**, *45*, 375303.

[12] H. Y. Jeong, J. Y. Lee and S.-Y. Choi, *Adv. Funct. Mater.* **2010**, *20*, 3912.

[13] E. Gale, R. Mayne, A. Adamatzky and B. de Lacy Costello, *Materials Chemistry and Physics.* **2014**, *143*, 524.

[14] M. H. Boratto, R. A. Ramos, M. Congiu, C. F. O. Graeff and L. V. A. Scalvi, *Applied Surface Science.* **2017**, *410*, 278.

[15] S. Ren, H. Qin, J. Bu, G. Zhu, J. Xie and J. Hu, *Applied Physics Letters.* **2015**, *107*, 062404.

[16] K. Tang, A. C. Meng, F. Hui, Y. Shi, T. Petach, C. Hitzman, A. L. Koh, D. Goldhaber-Gordon, M. Lanza and P. C. McIntyre, *Nano Lett.* **2017**, *17*, 4390.

[17] A. Regoutz, I. Gupta, A. Serb, A. Khiat, F. Borgatti, T.-L. Lee, C. Schlueter, P. Torelli, B. Gobaut, M. Light, D. Carta, S. Pearce, G. Panaccione and T. Prodromakis, *Advanced Functional Materials.* **2016**, *26*, 507.

[18] H. Tian, H. Y. Chen, B. Gao, S. Yu, J. Liang, Y. Yang, D. Xie, J. Kang, T. L. Ren, Y. Zhang and H. S. Wong, *Nano Lett.* **2013**, *13*, 651.

[19] N. Xiao, M. A. Villena, B. Yuan, S. Chen, B. Wang, M. Eliáš, Y. Shi, F. Hui, X. Jing, A. Scheuermann, K. Tang, P. C. McIntyre and M. Lanza, *Adv. Funct. Mater.* **2017**, *27,* 1700384.
